# An Analysis of Power Dynamics Affecting Handwashing Interventions in Sierra Leone: Findings From a Qualitative Participatory Study

**DOI:** 10.2196/39226

**Published:** 2023-01-27

**Authors:** Hanna Luetke Lanfer, Nicola Brew-Sam, Constanze Rossmann

**Affiliations:** 1 School of Public Health Bielefeld University Bielefeld Germany; 2 Department of Health Services Research & Policy Australian National University Canberra Australia; 3 Department of Media and Communication Ludwig Maximilian University of Munich Munich Germany

**Keywords:** participatory approaches, health promotion, handwashing, power dynamics, Sierra Leone

## Abstract

**Background:**

Handwashing is an effective and cost-efficient health behavior for preventing infectious diseases; however, its practice is shaped by multiple contextual factors and inequalities between different social groups in Sierra Leone. To address these inequalities, participatory approaches that allow a more equitable distribution of resources and the development of locally tailored interventions are increasingly used. However, social power dynamics have not been well integrated into the concept of participation, despite their known impact.

**Objective:**

We sought to investigate the role of power dynamics in participatory approaches to handwashing in Sierra Leone.

**Methods:**

From a socio-ecological perspective, this qualitative, formative interview study aimed to identify relevant actors and their power relationships before designing a participatory handwashing project in rural Sierra Leone. A field experiment with focus groups and a research diary compared the development of power dynamics in a participatory, community-driven approach with that in a nonparticipatory top-down approach.

**Results:**

According to our formative study, in community-based projects, multiple groups and actors interact directly or indirectly with each other, located within a macro level (eg, political institutions), meso level (eg, community leaders and groups), and micro level (eg, families) of a socio-ecological model. Although distinct leadership structures were noticeable and affected intervention attendance and processes of change in nonparticipatory approaches, community-led activities and handwashing increased in the participatory approach, irrespective of the leadership structure. Despite their ambivalence, the strategic inclusion of different community leaders appeared essential to enhance the value of the project, mobilize creative action, and empower lower-ranking individuals to practice handwashing. A similar ambivalent role could be observed in relation to external researchers, especially if they come from a different cultural background than the research participants, for example, from a Western country in a non-Western project setting. Although external researchers can initiate a project or provide certain resources, distinct expectations regarding their roles and resources can impact participatory efforts and power relations.

**Conclusions:**

The results highlight the advantages of participatory approaches for health promotion. Power dynamics should be a core component of continuous reflection and analysis in participatory projects.

## Introduction

### Background

Handwashing is considered an effective and cost-efficient means of preventing transmission of bacterial and viral contamination associated with diarrheal and respiratory infections [1-3]. However, with an overall low prevalence of handwashing, especially in rural areas, diarrheal diseases and respiratory infections continue to be a leading cause of morbidity and mortality in Sierra Leone [4], making handwashing an urgent topic for public health.

Since long, researchers have pointed out that health-related behaviors such as handwashing are not only determined by individual behavior but also shaped by multiple contextual factors [5]. A recent formative study [6] found that factors in the social and structural environments influence handwashing practices in rural Sierra Leone. Although some factors affected all individuals in a community (eg, interrupted water supply and perceived social exclusion from accessing government support), other barriers were not experienced evenly, as power asymmetries between different social groups granted certain people privilege to access the material and immaterial resources necessary for handwashing (eg, information and decision-making power over financial decisions).

### Participatory Approaches in Health Promotion

Recognizing the relationship between health behaviors and social and structural inequalities, there has been a trend toward participatory approaches in health promotion. Participatory approaches build on the theory of empowerment by Freire [7,8] and a practice of genuine dialogue, in which different groups of people reflect on their experiences, their identity and structures of power, and oppression to transform their circumstances. By including different actors (eg, community groups, health professionals, policy makers, and researchers), participatory approaches have the objective of distributing power and resources more equitably, diversifying knowledge production of health issues, and developing actionable and sustainable solutions [7,9,10]. Despite these principles, the authors have argued that power dynamics have not been well integrated into the concept of participation and are neglected in the literature [11-13]. There are 2 aspects to this critique. On the one hand, the composition and constellation of groups in consideration of their different forms and expressions of power before and during participatory approaches have been discussed relatively rarely [12,14]. Overlooking the genuine inclusion and composition of relevant social groups in participatory approaches then bears the risk of contributing to the reinforcement or even exacerbation of existing inequalities to the detriment of inclusive dialogue and empowerment of those most vulnerable [15-18]. On the other hand, while participatory approaches have a history of success and have been shown to improve health outcomes of individuals and groups [19], they have increasingly become a buzzword and a requirement in project proposals, “often applied as a tick-box exercise” [13]. Amid its upsurge, authors have warned of the risk of tokenism, when community-based approaches (top-down projects that take place in the community) become relabeled as participatory approaches (bottom-up projects that are driven by the community) [20]. Top-down, paternalistic approaches may also implicitly increase existing power inequalities and silence those most vulnerable [10,13]. Moreover, they can overlook important behavioral determinants and leave existing social capital for communal action untouched [21].

### Objectives

Given the previously examined social and structural inequalities related to handwashing practice in Sierra Leone [6], it is worthwhile to further explore the roles of different groups and their unfolding power dynamics in participatory approaches versus nonparticipatory approaches [12,13]. Hence, the objectives of this project were 2-fold. First, we aimed to explore the constellation of relevant groups and their power relationships. Adopting a socio-ecological perspective, a framework that focuses on the reciprocal relationship between individuals or groups (micro level), their immediate (meso) and more distal influences (macro), and how they shape each other through interaction [22], we aimed to address the following research question (RQ):

RQ1: Who are the relevant groups at the micro, meso, and macro levels for handwashing projects in rural Sierra Leone, and what are their existing power dynamics?

Second, we aimed to compare how power dynamics unfold through participatory and nonparticipatory approaches and how they are related to handwashing outcomes.

RQ2a: How do group and power dynamics unfold throughout a community-based participatory versus nonparticipatory handwashing project?

RQ2b: How are these dynamics related to handwashing outcomes in participatory versus nonparticipatory groups?

## Methods

**Figure 1:**
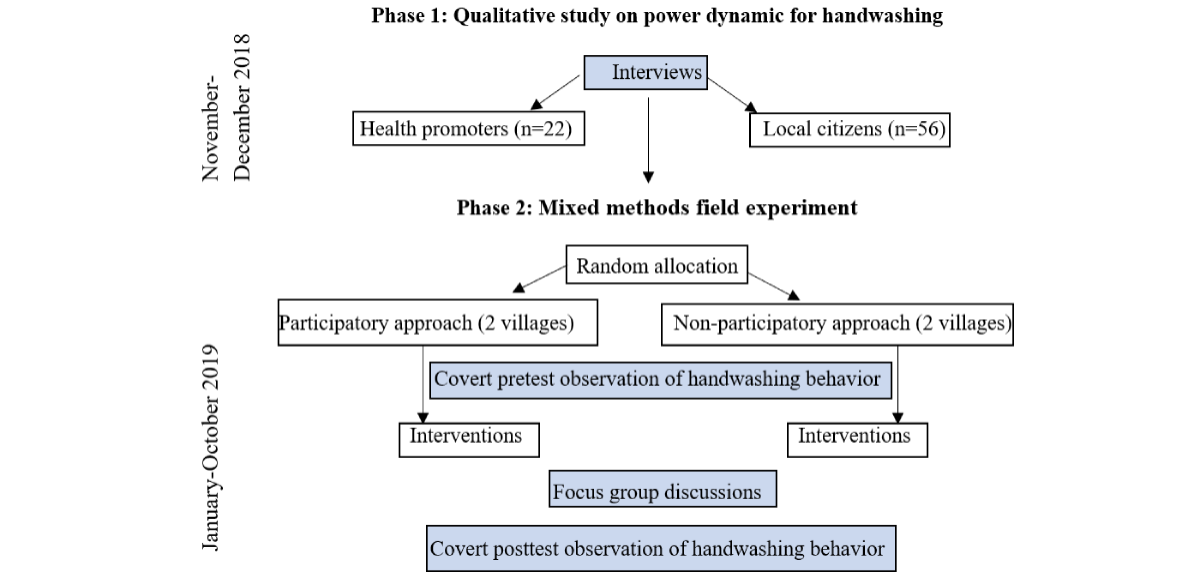
Flowchart of the study design.

This research project was carried out in 2 phases and used a formative study (November-December 2018) and a field experiment (January-October 2019; see Figure 1 for a flowchart of the study design).

### Formative Study

To inquire about the power dynamics relevant to handwashing (RQ1), semistructured interviews were conducted with health promoters (n=22) and local citizens (n=56) in Sierra Leone. Separate semistructured interview guides were developed for each sample to discuss community structures and power dynamics in participatory health projects.

#### Sampling and Procedure

To represent the relevant structures of health promotion in Sierra Leone, health promoters were purposefully recruited using the first author’s professional network consisting of research, government, and nongovernmental organization (NGO) partnerships established when working for the West African Ebola response and recommendations. Moreover, the Ministry of Health was contacted and asked for recommendations of interview partners. To recruit local citizens, contact persons in 8 different locations across Sierra Leone were identified. Working with 2 local research assistants, the research team traveled to the locations and asked for permission from the local chief. On consent from the chief, the contact person was asked to suggest 6 to 8 participants based on a list of criteria (ie, gender, different age groups, and fluency in the Krio language).

After providing informed consent, the health promoters were interviewed in English by the first author (14 at their workplace in Freetown, 6 in other parts of the country, and 2 via videoconference), whereas local citizens were interviewed at their homes by 2 local research assistants in the Krio language. The interviews lasted between 20 and 80 minutes (health promoters) and 15 and 35 minutes (local citizens), respectively. All interviews were audio-recorded. Health promoters did not receive any compensation for participation, and drinks and snacks were provided to the local citizens.

#### Data Analysis

All interviews were translated, transcribed verbatim, and fully anonymized. Qualitative content analysis [23] was used, starting with an initial coding frame built from the interview guide categories. All interviews were coded by the first author in a data-driven manner, and new subcategories were created, reviewed, linked, aggregated, and defined to ensure that they were mutually exclusive. During selective coding, the material was reviewed and recorded based on the final coding frame. Different stages of refinement of the coding frames were thoroughly discussed with the research assistants and coauthors.

### Field Experiment

#### Overview

A field experiment was conducted to compare how power processes unfold throughout participatory and nonparticipatory interventions and how they are related to handwashing outcomes (RQ2a and b). In addition to the treatment condition *participation*, another treatment was used; however, this will not be addressed in this paper. Further elaborations about the other treatment condition *communication approach* can be found in Luetke Lanfer [24]*.*

Four villages in northern Sierra Leone were randomly assigned to either the participatory or the nonparticipatory condition. A fifth village was recruited as the control group to receive no intervention. However, as this village received an intervention on hygiene by an NGO during the intervention period of this study and thus no longer represented an untreated setting, data were excluded from the analysis. A research diary and repeated focus groups (6 per intervention group) were used to document the power dynamics and processes of change related to handwashing in each intervention group. In addition, a pretest-posttest survey and observation of handwashing behavior accompanied the experiment. The results of these analyses are not presented in this paper and can be found in Luetke Lanfer [24]*.*

#### Sampling and Procedure

The field experiment was conducted over a period of 9 months, with a series of interventions from January to October 2019 (Table 1). A database of the first author’s former employer (NGO sector) identified 4 villages with access to a sustainable water source (borehole well) and with homogeneous characteristics (ie, approximately 700 residents, low socioeconomic status, and low prevalence of handwashing practice) in Bombali district. Once the chiefs agreed and provided informed consent on behalf of their villages, a community meeting was held to introduce the project to the whole village.

Except for a radio program with a physician from a government institution, all interventions were conducted face-to-face in the communities. Under the participatory treatment condition, community residents were actively involved in a series of interventions. The topic of each intervention (prepared by the research team) was discussed with the respective communities. For each intervention, volunteers from the communities joined to prepare and finalize the program, and later led it either independently or in collaboration with the research team. Community volunteers included representatives of different social groups, including community leaders, religious leaders, women, and young people, and cocreated intervention programs suited to their social groups, eg, a song creation workshop for women to either package handwashing messages in a memorable format (community P1) or to remind each other respectfully to wash their hands (P2). Moreover, a range of participatory activities such as small group discussions, roleplays, and call-ins during a radio show were used to reflect and discuss action points with community residents present during the respective interventions.

In the nonparticipatory condition, participants received lecture-type presentations of handwashing information presented by the research team or a medical worker without interactive elements. The content of the interventions in both conditions was based on materials provided by the government and our previous study [24], and it addressed handwashing knowledge, financial barriers to handwashing, and social and religious norms. Interventions in the nonparticipatory groups lasted between 30 and 60 minutes and up to 120 minutes in the participatory groups; they included preparatory meetings with the volunteers. Free meals were provided to participants after the interventions.

Focus groups were held a few days after each intervention, with up to 6 people participating in the preceding intervention. The discussions were held in the Krio language in the same location where all research-related activities took place, lasted between 15 and 25 minutes, were audio-recorded, and later transcribed verbatim.

A research diary was kept throughout the field experiment to document observations and occurrences during the official interventions. Each intervention group was visited at least twice a month for preparatory meetings with community-based messengers for the intervention meetings and the focus groups following each intervention. During or immediately after any community visit or phone call, descriptive notes were taken on paper or in a digital file; for example, to record the number of attendees in the interventions, the involvement of the community leaders, an increase in handwashing stations, and other events that appeared relevant to the dynamics in each of the groups. For each group, an individual digital text file was created, and all observations and memos were recorded in chronological order.

**Table 1:** Timeline and activities of the field experiment.

Month	Activity	Groups targeted
January 2019	Recruitment of 4 villages	Local leaders, letter from Ministry of Health
March 2019	Pretest survey and observation	N/A^a^
April 2019	First intervention (general community engagement meeting) and focus group discussion	Local leaders, nurse from government institution, village community
May 2019	Second intervention (radio show) and focus group discussion	Physicians from government hospital, village community
June 2019	Third intervention (religious congregations meeting) and focus group discussion	Religious leaders, Islamic and Christian believers
July 2019	Fourth intervention (group leader engagement meeting) and focus group discussion	Leaders from all community groups
August 2019	Fifth intervention (women engagement meeting) and focus group discussion	Women
September-October 2019	Posttest survey and observation; evaluation meetings	Village community

^a^N/A: not applicable.

#### Data Analysis

Data from the focus groups and research diaries were jointly analyzed using qualitative content analysis, similar to the procedure described for the interviews mentioned earlier.

#### Statement of Positionality and Composition of the Research Team

This study is based on data collected during the first author’s (HLL) PhD research project. HLL identifies as a White woman from a high-income country who worked in the humanitarian sector in Sierra Leone for 2 years before conducting this research. She speaks the local language Krio and was present in the participating villages for the presentation of the research project to the local communities, for all but 2 interventions, and for data collection. During the interventions in the field experiment, she stayed in the background and observed and took notes while they were led by 2 local researchers and research participants in the participatory condition. The second and third authors have similar cultural and educational backgrounds as HLL. They advised the first author throughout her research project and were only marginally involved locally in Sierra Leone.

Two local research assistants who contributed to data collection, community engagement, intervention planning, and implementation are not listed as coauthors in this manuscript on their own wish because of their lack of time to contribute to and review the manuscript. However, they have contributed to other publications related to this research and are listed as authors. Although we wished to include community residents in further research activities, including discussing the analyzed data, the outbreak of COVID-19 and the resulting travel restrictions in connection to technical challenges in establishing web-based meetings made this impossible. We acknowledge the contributions of the 2 local researchers and the study participants. This paper thus presents the views of a fairly homogeneous group, and we are aware that the findings presented here are influenced by our perceptions, worldviews, and social identities, which differ from the research participants.

### Ethics Approval

This study was approved by both the Sierra Leone Ethics and Scientific Review Committee and the Ethical Commission of Erfurt University (2018-0831).

### Consent to Participate

All study participants were informed about the project goals, topic, type of questions to be asked, and their right to decline participation or withdraw from the study at any time. Participants were asked if they had any questions before providing their informed consent. For illiterate participants, an impartial witness was present during the study purpose and procedures were explained. The participant thumbprinted the informed consent form in the presence of a witness, who then signed the consent form. Each participant was provided a copy of the consent form. All procedures were performed in accordance with the ethical standards of the 1964 Declaration of Helsinki and its later amendments.

## Results

### Samples

The participant characteristics of the formative interview study (N=78) are displayed in Tables 2 and 3. Table 4 provides an overview of the characteristics of the 4 intervention communities used in the field experiment.

**Table 2:** Professional background of health promotors (n=22).

Code	Participant’s professional background	Gender	Sector
HP1	International program manager	Male	NGO^a^
HP2	Local community engagement officer	Male	NGO
HP3.1 and 3.2	Local nurse	Female (both)	Health care
HP4	Local religious leader	Male	Local leadership
HP5	Local trainer of CHWs^b^	Female	NGO
HP6	Local community engagement officer	Male	NGO
HP7	International journalist	Male	Media
HP8	Local paramount chief	Male	Local leadership
HP9	Local religious leader	Male	Local leadership
HP10	Local policy maker and program manager	Male	NGO
HP11	Local policy maker and program manager	Male	NGO
HP12	Local journalist	Male	Media
HP13	Local religious leader	Male	Local leadership
HP14.1 and 14.2	Local community engagement officer	Male	NGO
HP15	Local program manager	Male	NGO
HP16	Local nurse	Male	Health care
HP17	Local-government official, national level	Male	Government
HP18	International media producer	Male	Media, NGO
HP19	Local-government official, district level	Male	Government
HP20	Local media producer	Male	Media, NGO

^a^NGO: nongovernmental organization.

^b^CHW: community health worker.

**Table 3:** Demographics of local citizens (n=56).

Characteristics			Value, n
**Sex**			
	Female	28	
	Male	28	
**Location**			
	Rural	28	
	Urban	28	
**Education**			
	No formal education	31	
	Primary school	14	
	Secondary school	10	
	College or university	1	

**Table 4:** Overview of characteristics of the 4 selected villages.

Intervention groups				Participatory 1		Participatory 2		Nonparticipatory 1		Nonparticipatory 2
Inhabitants (approximate), n				530		870		570		800
**Physical environment**										
	Electricity			No		No		No		No
	House-pumped water			No		No		No		No
	Number of borehole wells			1		2		1		1
	**Number of handwashing stations**									
		Private	0		0		0		0	
		Public	0		0		0		3	
	Number of latrines			7 shared by village		30 shared by village		1 per household		1 for 3 houses
	Cooking and eating places			Shared		Shared		Shared		Shared
	Community center			Yes		Yes		No		Yes
	Distance health facility			8 miles		5 miles		4 miles		2 miles
**Social environment**										
	Literacy rate			Low		Low		Low		Low
	Main occupation			Farming		Farming		Farming		Farming
	**Main gender roles**									
		Men household head	Yes		Yes		Yes		Yes	
		Women in charge of the household	Yes		Yes		Yes		Yes	
	Main religion			Muslims		Christians		Muslims		Muslims
	Weekly religious gatherings			Yes		Yes		Yes		Yes
	Leadership and CHW^a^			Active CHW, chief and leaders support project		2 active CHWs, chief and leaders support project		Active CHW, chief absent, few leaders support project		CHW very quiet, chief supports project, few leaders
**Media and communication**										
	Phone network coverage			Low		Low		Good		Average
	Radio reception			Good		Good		Good		Good

^a^CHW: community health worker.

### RQ1: Power Dynamics Relevant for Handwashing Behaviors

#### Overview

**Figure 2:**
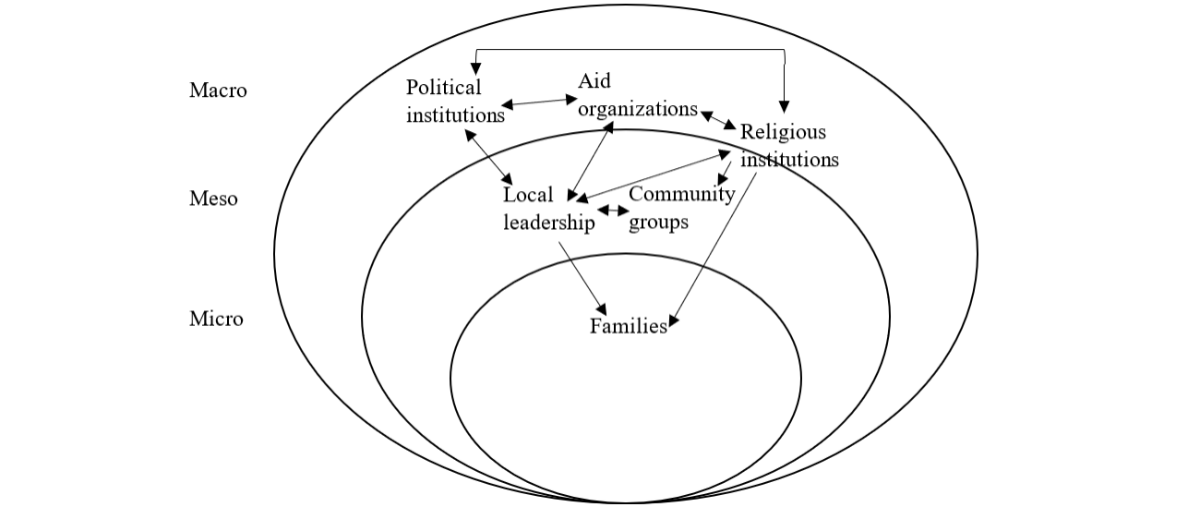
Loci of power on micro, meso, and macro levels.

Analysis of the interview data of the health promoter and local citizen samples yielded insights into hybrid, multilevel loci of power relevant for a community-based participatory approach in Sierra Leone. Multiple groups and actors interact directly or indirectly with each other, each of which is described briefly in subsequent sections and is located within the macro, meso, and micro levels of a socio-ecological model (Figure 2). The supporting data excerpts for each locus of power are listed in Table 5.

**Table 5:** Different loci of power in a socio-ecological model^a^.

Level, locus of power, and theme				Sample citations
**Macro level**				
	**Political institutions**			
		Facilitator of health projects	“Government is doing a lot, promoting and spearheading a lot of activities these days. This can also help with the credibility.” [HPM15]	
		Lacking connection with communities	“We are asking Papa Government... we wait for them to let them help and supply us.” [LCF4] “We don’t trust the government too much here. We trust our own people more.” [LCF4]	
		Limited executive power in communities	“For the government to succeed in most of their laws, they’ll have to communicate those laws to local leaders and encourage local leaders to encourage their people to like respect the laws, you see.” [HPM14.1]	
	**International and national aid organizations**			
		Financial support	“We need the support of partners to complement our activities because you know, the Ministry of Health and Sanitation hasn’t got all the resources it requires to carry out the activities. So that’s why... we bring in these NGOs and others.” [HPM19]	
		Different concepts of health and project implementation	“Some of my colleagues are not happy about this [being asked to cooperate in health projects] and some of them told me that the White man has indoctrinated me.” [HPM4]	
	**Religious institutions**			
		Effective distribution of health messages	“Whenever a message is being passed from the religious leaders, the people receive it. Because there are people in the community who don’t attend [community] meetings and even if they attend, they will not wait to listen, especially if the meeting doesn’t include sharing money. But if he goes to the mosque and the Imam is sharing the message, he will have patience.” [LCM6]	
		Assumed divine power	“And religion, I cannot overemphasize, the importance, the authorities should take these issues very important....I mean how to control these religious people. They have become so powerful that people believe in them more than God himself.” [HPM7]	
**Meso level**				
	**Local leadership**			
		Trust in locally known leaders	“We trust our chief and our leaders, because anything that happens outside, they will tell us and make it happen for us.” [LCF7]	
		Executive power in communities	“They [local leaders] also have the power over the community. Communities have their structures and whatever has been agreed upon by the leadership of the community and anyone violates it, there will be a penalty. The Ministry of Health is not able to institute penalty, but if the community leaders agree, they have their structures and they can make sure people obey.” [HPM11]	
		Influence over all community groups	“They [local leaders] are able to take decisions and people follow. Sometimes even when they are not doing the right things, people don’t have an alternative, they need to follow.” [HPM6] “We cannot operate well if we don’t have the backing of the community, and the chiefs are the key to the community.” [HPM16]	
		Gatekeepers of community members	“When we go for interventions and want to select community members, we say to the community leaders, ‘Can you send us the names of people that you want us to work with or train?’...the community leaders will give the names of men... the women often don’t fulfil the criteria.” [HPM11]	
	**Community groups**			
		Active participation in communal life	“Everyone is a member of one group at least: the youth group, the women’s group, the development group. So, we help each other out.” [LCM8]	
		Fear of an abuse of resources	“You know, many people like to take advantage. So, I prefer to leave things within the family. But if there is a community project, there should be accountability.” [LCF2]	
**Micro level**				
	**Families**			
		Tight relationships	“Individuals are surrounded by family and the relationship and trust at the family level – you can look at it as a positive environment, an enabling environment where action can take place.” [HPM10]	
		Low autonomy of lower-ranking family members	“You have mothers who want to participate, but maybe their mothers-in-law don’t want them or their husbands will say ‘No’. So that is why...you have to bring the family, you have to bring in the family and the community, so that you have that critical mass.” [HPM10]	

^a^For reporting results, participants from the sample of health promoters (HPs) and local citizens (LCs) received a code based on a number and their gender (ie, F for female and M for male); for example, HPM1 or LCM5.

#### Macro Level

Political and religious institutions and aid organizations were frequently mentioned as groups external to local communities, yet important in initiating, funding, and implementing participatory health projects. However, owing to being “community outsiders” and delivering varying degrees of direct involvement, these institutions or organizations were often viewed ambivalently.

##### Political Institutions

At the macro level, the Ministry of Health and Sanitation on behalf of the government is in charge of the health care sector and any health promotion activity. On one hand, the government’s authority in health-related projects was widely acknowledged and viewed as a facilitator, as they could occasionally make funds available or, by approving projects or initiatives, increase their credibility. On the other hand, local citizens also described a lack of connection between themselves and governmental institutions, including a lack of medical facilities, concomitant with a perceived inability to make their needs known, and distrust toward this largely unknown authority. The limited executive power of the government that was in charge of passing new laws but relied on other forces for their implementation was a frequently mentioned theme among the health promoters.

##### International and National Aid Organizations

International and national organizations (eg, foreign government agencies, intergovernmental organizations, and civil societies) were described as another ambivalent group. Their financial role in funding programs and their implementation have been highlighted by numerous health promoters and local citizens. Amid the lack of available national funds, international organizations have especially been said to enable the development and implementation of health programs. However, in the case of international bodies, White hegemony in a postcolonial country, different understandings between biomedical and spiritual aspects, traditional concepts of health, culturally ill-fitting implementation, and a lack of involvement of local stakeholders were mentioned to affect foreign sponsorship of and involvement in local programs.

##### Religious Institutions

Among cultural and social institutions, religious bodies, specifically Islamic and Christian faith communities, were frequently mentioned as relevant for health promotion. Owing to the perception of representing God’s word, religious leaders were considered highly trusted and influential in every layer of society and were thus located between the macro and meso levels (Figure 2).

In the past, most health promoters had cooperated with faith-based institutions, and this strategy appeared to be well-known among local citizens. Both samples described religious institutions as being effective in disseminating health messages and implementing projects. Although local citizens and some health promoters framed the power of religious leaders as only positive for health promotion, their assumed authority as a divine power was also viewed as risky if religious leaders were granted too much power.

To summarize, despite power on a macro level sometimes being felt “far from” the local individual, decisions on a macro level impacted health projects on a local level.

#### Meso Level

At the meso and community level, local leaders and community groups were identified as essential loci of power whose involvement was required for any nonparticipatory project.

##### Local Leadership

The local leadership group stood out as the most mentioned locus of power and, therefore, appeared to be crucial for health promotion. For local citizens, their leaders were described as more approachable than government officials because of their presence in the community and as they were largely trusted as to having the good of the community in mind. Local leaders were also described as possessing the executive power that the government did not have at a community level; for example, implementing laws and issuing penalties in case of violations. Being at the top of strongly hierarchical community structures, they influenced all lower-ranking residents, and thus either perpetuated the status quo or enabled change. With regard to community projects, various health promoters described the gatekeeping of local leaders as challenging, as women in particular were often neglected in project participation.

##### Community Groups

Community relationships and social capital are based on a dense network of community groups in which residents are members and thus actively participate in communal life. In addition to their usual mandate, community groups also perform extracurricular activities; for example, joint infrastructure improvement coordinated by local leaders. However, approximately half of the local citizens described restraints toward engaging resources in communal projects because of fear of abuse.

In contrast to the macro level, the meso level was reported to have a direct impact on projects in local communities.

#### Micro Level

Families were allocated as the main locus of power at the micro level, and their dynamics and hierarchical relationships could impact participation in health promotion.

##### Families

Although family networks were mentioned as tight and members took care of each other, the hierarchical structures within families were also seen as obstacles. The age and gender gradient, for instance, prevented particularly younger women from getting engaged in community activities independently of their family’s approval.

To summarize the 3 levels, our analysis showcased multiple groups that interacted dynamically and impacted local projects in community settings (Figure 1). Although political institutions and aid organizations were external to the communities, they often initiated health projects and shaped their development through decisions regarding their implementation. Religious institutions have a dynamic influence at the macro and meso levels, with influence over various loci of power. Finally, local leaders appeared central to participatory projects, as they interacted with forces across different levels. For participatory approaches to be successful, all groups must be involved in the intervention planning and fieldwork, especially the community leaders, to facilitate access to the less powerful.

### RQ2a: Group and Power Dynamics Unfolding Throughout an Intervention

#### Overview

During the field experiment, the roles of the different actors and processes of change were observed and compared. This section describes the roles of different actors throughout the intervention and observed power processes in the participatory (villages P1 and P2; P=participatory village) compared with the nonparticipatory (villages NP1 and NP2; NP=nonparticipatory village) conditions related to intervention outcomes.

In the present case study, local leaders were influential for intervention attendance and for processes related to implementing behavior change regarding handwashing; they are thus the focus in this section. Moreover, although data were gathered on all actors, community groups and families were hardly mentioned and are thus not described here.

#### Local Leaders

The leadership structures differed in each village with two main differences: (1) the number of active leaders and (2) whether the group of leaders was unified or divided throughout the intervention. Although village P1 was characterized by a small group of divided leaders, P2 displayed a large, unified leader group. NP1 demonstrated a large group of divided leaders, whereas NP2 was characterized by a small, unified leader group. P2 (large active leader group) and NP2 (small unified leader group) had groups of leaders that were supportive of the handwashing project and were majorly present during the interventions. In both groups, if the chief could not attend an intervention, he always sent a representative who endorsed the meeting at the start. In conversations with different leaders in both groups, it was remarkable how important they considered their attendance in such events, as it would increase the perceived importance and attendance of other community members:

The big people in this town also come to this program and they say that handwashing is important. This helps because sometimes... other people do not agree.

Female participant, NP2

In the 2 communities with unified leader groups (P2 and NP2), community members spoke highly of their leaders. In contrast, the most distinguishable leadership group and community dynamics were found in NP1 (a large group of divided leaders), with the lowest number of intervention participants noted overall (as few as 5 participants). The chief was absent most of the times, and various disagreements could be recorded, including physical and verbal conflicts:

During the first contact with the community when we presented the research project, a group of about 10 people was present, yet not the chief. When project information was handed over to a male participant who had introduced himself as the chief’s representative, a heated discussion started whether he was the appropriate person to take the documents. Two men left the scene shouting and no agreement could been found.

Observation notes, NP1

In P1 (a small group of divided leaders), the absence of the chief was as noticeable as it was in NP1. Moreover, 3 conversations were overheard, during which community members criticized the lack of involvement of the chief in community affairs. However, interventions in P1 were still highly frequented, which can largely be credited to the few, very active local leaders (eg, community groups and religious leaders) who mobilized people to join the intervention meetings.

#### Health Care Institutions and Actors

In all villages, participants asserted that representatives from the health care sector raised the credibility of the project and the roles of other actors and their messages:

We don’t know him [doctor] personally, but I know the hospital and it’s a good hospital... So, I trusted him.

Female participant, NP2

#### Research Team

It became apparent that the concept of conducting research was not well-known in any of the villages. During the first months of the project, the first author, representing access to NGOs and other funding agencies in the eyes of the communities, was expected to make funds for infrastructure projects available or to enable connection to funding partners:

After the intervention, a group of village elders in P2 approached me, saying we couldn’t do a project without supplying the necessary materials for handwashing... I expressed my understanding and explained that the focus of the research project was to develop solutions from within the community. Similar requests and mentions of “unmet responsibilities” were also noted in the other villages.

Observation notes, P2

With time, these requests became fewer, and the presence of the research team was also described as a reminder for handwashing and encouraging community action in all villages, except NP1 (large group of divided leaders):

Every time you came, people were saying “Oh, let us wash our hands” and we were getting used to it. So, we want you to do the same for us for other sicknesses, like malaria, because we have seen better health.

Male participant, NP2

People started saying, “They [researchers]...have an interest in our town” and they also started to see the work you are doing and the knowledge we are getting. I think...handwashing has become a sustainable practice.

Male participant, P2

Spending time in each village, even beyond the interventions (eg, eating together or the 2 local researchers praying in the village mosque on Fridays), was viewed as important and key to building relationships with outsiders in a community-based research project.

#### Religious Leaders

The larger proportion of the village residents in our sample was identified as Muslims, for whom handwashing is a part of the 5 daily ablutions. Thus, their role was to enhance the importance of handwashing in all villages:

The Imam told us about the daily prayers and why we do it. He also told us about cleanliness of our body and how God sees a clean person....I will make sure to wash my hands for prayers and for cleanliness.

Male participant, NP1

Moreover, as religious activities throughout the week were generally attended by all community members at least once, religious leaders were acknowledged for being able to reach out to everyone, including community leaders:

I believe you didn’t make a mistake in choosing these people [religious leaders] and letting them be part of the counsellors... They can counsel everyone, from the leaders to the stubborn people.

Male participant, P1

Overall, interactions among the 4 loci of power were noticeable. Local leaders were the most influential factors for intervention attendance and measures to change behavior. Distinct leadership structures were observed in all villages, with consequences for intervention attendance and community-led activities. The most distinct structures were observed in P2, with a large group of active, unified leaders, and in NP1, with divided leaders who largely refrained from participating or even obstructed intervention activities. In addition, there were great overlaps in all villages regarding the perception of 3 other external actors—health care workers, the research team, and religious leaders—who contributed to increasing the credibility of the project and encouraging community-led activities. A lack of interaction with the community was mentioned in the nonparticipatory condition; in contrast, the participatory condition appeared to foster community-led activities in an already active setting or compensate for divided leadership.

### RQ2b: Handwashing Outcomes in Participatory and Nonparticipatory Approaches Related to Power Dynamics

This section assesses the different processes of handwashing behavior change between participatory and nonparticipatory villages from a qualitative research perspective.

After the second intervention, the first internal community-led activities (independent of the interventions) were noted in participatory villages. Thus, the participatory approach led to the continuation of the initial activities. For instance, at some point in the past, biweekly community group meetings were held in P2 (a large group of unified leaders) to discuss relevant health topics, but they were discontinued, as nobody felt responsible for facilitating them. With a new schedule, participants in P2 established 2 new groups: one for women and one for the general population, which were used to discuss current health issues and develop action points. P2 was the only intervention group in which communication about health topics was publicly organized compared with the other villages that reportedly reached out spontaneously to others who had not attended the previous intervention. Overall, after the first 2 months, questions tended to subside from financial barriers to more practical matters in the later stages of the intervention period, and community members increasingly responded to each other’s questions, primarily in the participatory condition but also to some extent in the nonparticipatory groups:

A man said whenever he went to his farm, he faced difficulties to wash his hands before eating his lunch as he had neither water nor soap. Various people offered suggestions, e.g., that he could take along an extra water bottle.

Observation notes, NP1

In the participatory groups, a huge proportion of the interventions were interactive and thus had space for questions and discussions. Although participants said they were unfamiliar with interactive activities at the start of the project, they increasingly described them as being an advantage for obtaining more ideas and opinions, making health interventions enjoyable and encouraging the development of local solutions:

This idea of a tippy-tap was very interesting. It made us think about what we can do for ourselves... Look at all the people who do handwashing now – we are proud of what we have achieved.

Male participant, P2

In the nonparticipatory groups, there were quests for more participation, most notably in NP2 (a small group of unified leaders):

The chief and his representative told us after the intervention that they wanted us to make it more interactive and involve the audience.

Observation notes, NP2

An increase in new handwashing stations was noted in all villages in July and August (the last 2 months of the intervention period). However, it was noticeable that in the 2 participatory groups (P1 and P2), overall, more fully functioning handwashing stations were equipped with water and soap. Both participatory villages had also assigned designated community members for each station who were in charge of caring for it, a process that was not mentioned in the nonparticipatory groups.

**Figure 3:**
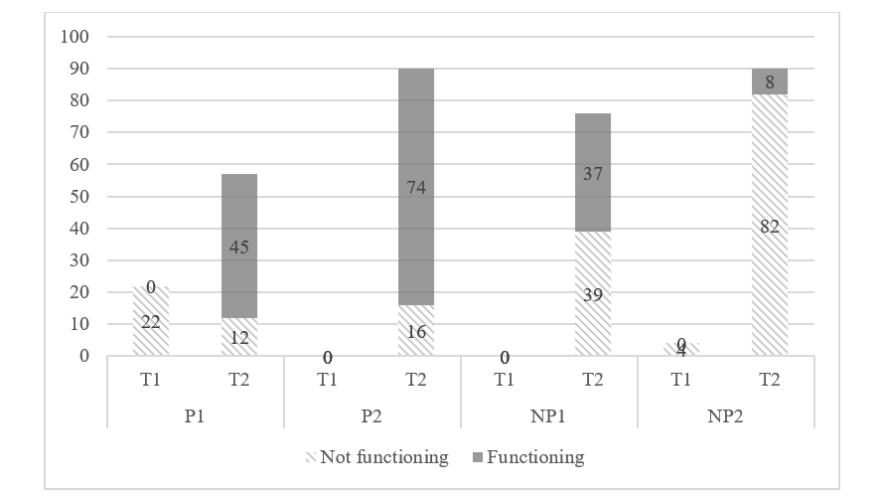
Functioning and not functioning handwashing stations before and after the intervention period (N=339), with n=26 for T1 (pretest) and n=313 for T2 (posttest).

In the participatory groups, participation appeared to have either increased communal activities in an already active setting (P2, a large group of unified leaders) or initiated some activities despite the absence of united leaders (P1, a small group of divided leaders), resulting in a considerable increase in the functioning of handwashing stations. However, it should be noted that steps toward increasing handwashing practices were also made in the nonparticipatory groups to a different extent and at a different pace (Figure 3).

Taken together, our analysis throughout the field experiment enabled conclusions about the processes and power dynamics in participatory versus nonparticipatory approaches related to handwashing outcomes. Although overall more functioning handwashing stations—an indication for behavior change—were found in the participatory condition, intervention outcomes were also shaped by influences independent of the treatment condition. In this way, distinct leadership structures stood out and delivered important insights into how these affected the way handwashing behavior change was implemented by different villages. For the acceptance of a research project that does not come with a large budget for new infrastructure and is initiated by individuals outside the villages, including a White person, and their meaning in postcolonial, aid-receiving countries, cocreating interventions with community members, valuing local knowledge and capacities, and spending considerable time to build relationships with different social groups was important to gain acceptance and continued participation in the project.

## Discussion

### Principal Findings

Our study applied a comprehensive methodological approach using a formative interview study and a field experiment that used qualitative data collection methods. By using a socio-ecological model, our formative study systemically conceptualized the relationships between different loci of power before a community project [13]. Our study thus contributes to discussions about the meaningful engagement of different actors in participatory health projects [25,26]. The results indicated that local leaders are closely related to all other loci of power and can be gatekeepers or facilitators of their communities; for this influence, their involvement should be central to participatory approaches.

The consecutive field experiment used 2 qualitative methods to understand the processes of change and development of power dynamics in different villages in relation to the treatment conditions (participatory vs nonparticipatory approaches). Combining the 2 methods allowed for a better understanding of how behavior change occurred and how it was affected by influences independent of treatment conditions. The qualitative results indicated that P2 stood out with regard to its leadership structures and how these supported intervention attendance, community-led activities, and handwashing practice in comparison with the leadership groups of the other villages. The results provide important insights into the influence of community and leadership structures and the loci of power on intervention outcomes, as also shown by Mkandawire and Hendriks [27]. Our qualitative findings were confirmed by quantitative statistical analysis of a pretest–posttest survey and observation, as described in Luetke Lanfer [24].

Using a solution-focused perspective, our intervention design did not attempt to work against or exclude powerful actors but rather included them strategically for the empowerment of lower-ranking community members. Studies have shown that in patriarchal societies, such as Sierra Leone, women experience gender-related boundaries to enact health behaviors [6,28], and participatory approaches can contribute to the empowerment of rural women [29]. Our findings provide some indications in this direction, with a large number of women attending the interventions. Our results also showed that a participatory approach can compensate for inactive leadership (P1) and is thus crucial for community interventions. According to the number of functioning handwashing stations counted before and after the interventions, more functioning stations were noted in the participatory than nonparticipatory villages. The success of the participatory approach resonates with other experiments that used participation as a treatment condition [30,31].

Even though the research team had aimed to stay in the background during community activities, their supporting or hindering role in a participatory community project was crucial in addition to local leadership and community structures, as authors have questioned the legitimacy of foreign researchers in heterogeneous social settings and their potential impact on reinforcing existing inequalities, especially in settings with White, Western researchers in former postcolonial, aid-receiving countries such as Sierra Leone [32,33]. This requires ongoing reflection, particularly from those who uphold power and benefit from historical privileges. Although a combination of different external actors appeared to have a positive impact on the project, the presence of the research team and their lack of financial resources caused some discontent at the start but was later described more positively. Further research is needed to examine power inequalities and related tensions arising from the involvement of research teams in local participatory community projects and to find locally meaningful solutions for such tensions. In our project, managing some of these tensions required frequent presence in all villages, showing an interest in communal life, and valuing local traditions and customs.

### Limitations

This study has several limitations. First, research was conducted in rural Sierra Leone and thus provides a snapshot of the loci of power for a participatory project in this particular context only. Although our results might not be generalizable, our analytical approach during our formative study (using a socio-ecological model to allocate different groups and their power relationships on different levels) can be recommended before designing participatory projects. Second, selection bias might have affected the views portrayed by the participants in our formative study. For the expert interviews, the first author’s network was used as a starting point for recruitment efforts. Although this network stretched across different sectors (eg, media, NGO, and religious institutions), it might have limited diversity among the study participants. For the focus group participants, chiefs in each setting were asked for recommendations, which is customary in Sierra Leone. By giving the chief a list of criteria, we aimed to recruit heterogeneous groups with different backgrounds, but a bias cannot be excluded. Third, we collected considerable amounts of data on certain actors (eg, local leaders) during the field experiment because of the high presence of these actors in the focus group discussions. Considerably fewer insights were gained on the roles of community groups or families, and they might have been counteracted by separate focus groups for leaders and ordinary community members in future studies. Fourth, the leadership structures could only be included in qualitative analyses to a limited extent, and follow-up research (including quantitative analyses) is needed to investigate the association between leadership structures, nonparticipatory approaches, and intervention outcomes. Fifth, though research activities were jointly carried out and analyzed with local research assistants, they did not contribute to writing this paper owing to other commitments. The inclusion of village communities in data analysis and publishing would have been desirable, but travel restrictions during the COVID-19 pandemic did not allow this. Therefore, the views and perspectives presented in this paper should be viewed as shaped by the cultural, historical, and socioeconomic background of the first author, a White woman and an external researcher to Sierra Leone.

### Conclusions

Although participatory approaches to health promotion have increased in popularity, the conceptualization of power dynamics between different actors has gained less attention. To address this gap in the literature, we initially conceptualized different loci of power relevant to participatory approaches in Sierra Leone by using a socio-ecological framework. These findings were then translated to an intervention design for a field experiment on hand washing. Using participation as a treatment condition, the relationships between different actors and the effects of treatment conditions were investigated by combining 2 qualitative methods. The findings indicated that the participatory condition had a greater impact on handwashing behavior change and increased community-led activities. The dynamics between different loci of power and their impact on community participation in participatory projects require continuous reflection to decrease the potential for reinforcing structural inequalities.

## Abbreviations

NGO: nongovernmental organization
NP: nonparticipatory village
P: participatory village
RQ: research question

## Data Availability

The (anonymized, nonidentifiable) data sets used or analyzed during this study and additional information on the methodology are available from the corresponding author on reasonable request.
